# A Retrospective Study on the Outcome of Coronavirus Disease 2019 (COVID-19) Patients Admitted to a District General Hospital and Predictors of High Mortality

**DOI:** 10.7759/cureus.53432

**Published:** 2024-02-01

**Authors:** Zahid Khan, Gideon Mlawa, Saiful Islam, Suhier Elshowaya, Mohammad Saleem

**Affiliations:** 1 Acute Medicine, Mid and South Essex NHS Foundation Trust, Southend-on-Sea, GBR; 2 Cardiology, Barts Heart Centre, London, GBR; 3 Cardiology and General Medicine, Barking, Havering and Redbridge University Hospitals NHS Trust, London, GBR; 4 Cardiology, Royal Free Hospital, London, GBR; 5 Internal Medicine and Diabetes and Endocrinology, Barking, Havering and Redbridge University Hospitals NHS Trust, London, GBR; 6 General Medicine and Gastroenterology, Barking, Havering and Redbridge University Hospitals NHS Trust, London, GBR; 7 Internal Medicine, Barking, Havering and Redbridge University Hospitals NHS Trust, London, GBR

**Keywords:** risk factors for covid-19, covid-19 mortality predictors, risk of covid-19 mortality, coronavirus disease 2019 (covid-19), covid-19 outbreak

## Abstract

Background: The clinical features and severity of coronavirus disease 2019 (COVID-19) vary between patients and countries. Patients with certain conditions are predisposed to poor outcomes compared with those without medical conditions, such as diabetes, dementia, and hypertension (HTN).

Methods: The aim of this retrospective study was to assess factors associated with higher mortality in patients with COVID-19 infections and to identify the reason for hospital admission in these patients. The study was performed on patients admitted between 1 and 31 March 2020. Data collection was done retrospectively from electronic medical records.

Results: There were 269 patient admissions during this period, of which 147 were included in this audit. The mean age of COVID-19-positive patients was 62.8 years and 65.9 years for COVID-19-negative patients during this period. Forty-seven patients requiring hospital admission were COVID-19 positive and 93 were COVID-19 negative. There were no COVID-19 swabs in the seven patients included in the audit. Approximately 50% of the COVID-19-positive patients presented with fever and shortness of breath (sob), followed by dyspnea and cough (seven patients). The most common comorbidity was HTN, followed by type 2 diabetes mellitus (T2DM) and ischemic heart disease (IHD). The survival rate was 72.3% in COVID-19-positive patients and 80% in COVID-19-negative patients. The average length of stay was 14.4 days for COVID-19-positive survivors compared to 7.8 days for COVID-19-negative survivors. Most patients who tested positive for COVID-19 infection received oseltamivir vaccination and antibiotics. The presence of HTN, diabetes mellitus (DM), age, and organ failure was associated with a high mortality risk.

Conclusion: Our study supports the findings of previous studies that diabetes, HTN, coronary artery disease, old age, and organ failure were associated with high mortality in patients admitted to hospitals with COVID-19 infections.

## Introduction

The coronavirus disease 2019 (COVID-19) pandemic that started in China and spread rapidly worldwide continues to cause occasional outbreaks in various parts of the world. The manifestation can vary from minor asymptomatic illness to fulminant systemic inflammatory syndrome unleashed by a cytokine storm [1]. There is some variation in the data for COVID-19 morbidity and mortality across various regions due to underreporting in some regions because of poor health infrastructure [1,2]. The World Health Organization (WHO) reported a significant difference in the outcomes of patients with COVID-19, and the case fatality rate was far higher in certain parts of the world compared to others. The fatality rate reported in South Europe and the United States is significantly higher compared to China and North Europe [2]. Similarly, the case fatality rate in Italy was far higher than in China and was linked to male sex, older age, and comorbidities [2]. Other studies have highlighted similar findings and have attributed advanced age, male sex, and comorbidities such as diabetes mellitus (DM), obesity, hypertension (HTN), renal disease, coronary artery disease (CAD), and malignancy to poor outcomes in patients with COVID-19 infections [3-8]. The spectrum of presentation can vary from asymptomatic infection to lower respiratory tract infective symptoms, such as cough, shortness of breath (sob), fever, sore throat, anosmia, and acute respiratory distress syndrome in severe cases [9].

Several studies have investigated mortality predictors in patients with COVID-19 pneumonia and identified age, sex, cardiovascular and metabolic abnormalities, and C-reactive protein (CRP) as significant prognostic factors [10-12]. Genetic factors may play a role in the progression of acute respiratory distress syndrome severity in patients with COVID-19, whereas the opposite effects were found with blood group types. Blood group type A has been associated with a higher risk of disease severity or progression, while blood group O was found to exhibit protective effects [10,13]. After the initial decline in the number of cases, there was an increase in the number of patients requiring hospital and intensive care admission in parts of the European Union and European Economic Area (EU/EEA) countries, attributed to the Delta variant (B.1.617.2) and the Omicron variant (B.1.1.529) and inadequate vaccination in some of these countries [14]. The mortality rate of the severe form of infection varies among studies, and the rate is higher in patients admitted to intensive care units (ICU), with rates ranging from 8.1% to 30% for hospitalized patients in medical wards and 16% to 78% for patients admitted to ICU [9].

Several biomarkers, such as high neutrophil-to-lymphocyte ratio, high creatinine, lactate dehydrogenase (LDH), direct bilirubin, alanine aminotransferase (ALT), D-dimer, CRP, serum ferritin, interleukin-6 (IL-6), and procalcitonin (PCT), were associated with a higher mortality risk in patients with COVID-19 [1]. Most patients had mild infections without requiring hospitalization; however, 5-10% of patients developed pneumonia after a week of infection requiring hospitalization. Only a small number of patients develop further complications requiring intensive care support, with a mortality rate of approximately 1.4%. The risk of severe adverse outcomes was associated with an increasing number of comorbidities and patients aged ≥60 years, and underlying comorbidities such as cardiovascular disease, DM, chronic respiratory disease, malignancy, immunodeficiency, obesity, and male sex were associated with a higher mortality risk [15]. This study aimed to identify factors, including comorbidities and features of COVID-19 infection, associated with high mortality in patients with suspected COVID-19. The data is presented in tabular and graphical forms, and comparisons are made with other national and international studies.

## Materials and methods

Methodology

This was a retrospective audit of patients presenting to Queen's Hospital, Barking, Havering and Redbridge University Hospitals NHS Trust, in Romford with suspected COVID-19 requiring admission. The audit was approved by the Audit and Research Department of Queen's Hospital, Barking, Havering and Redbridge University Hospitals NHS Trust (approval number: 074-21), and access was granted to patients' medical and electronic records. Patient confidentiality was maintained during this process, the collected data was saved on hospital local computer drivers, and access was granted to all the researchers involved in the data collection

Aims and objectives

The primary aim of this audit was to determine associated symptoms, reasons for admission, factors associated with a higher chance of hospital admission, and predictors of poor outcomes. We aimed to assess factors associated with poor outcomes in patients admitted with COVID-19 and to compare our findings with published national and international data. The audit was approved by the local audit and research departments. We collected data for patients admitted to the hospital between 1 and 31 March 2020 and presented local findings, and data collection was performed by three participants. The final data were reviewed by two reviewers for any discrepancies, and data were excluded or included after discussion between the two independent reviewers based on the inclusion and exclusion criteria.

Inclusion and exclusion criteria

The inclusion criteria were patients aged >18 years, suspected to have COVID-19 based clinically and confirmed biochemically or radiologically, and requiring hospital admission. The exclusion criteria were patients aged <18 years, those admitted for surgical reasons, and those who were not suspected to have COVID-19. Patients with missing data were excluded from this study, and tremendous efforts were made to retrieve data for all patients; however, patients with missing data despite our maximum efforts were excluded from the study to minimize the risk of selection bias. This was unanimously agreed upon by all researchers involved in the study.

Data collection and analysis

Data were collected from the patients who met the inclusion and exclusion criteria. The collected data included patient demographics, presenting problems, and medical histories. Patient records were retrieved from patients' medical notes, and biochemical and radiological test results were retrieved from the electronic data system. We collected data on patient demographics, length of stay, blood cultures, flu swabs, administration of antibiotics or antiviral therapy, COVID-19 swabs, mortality data, past medical history, and factors associated with high mortality risk. A significant number of patients had missing data and were excluded from the audit, resulting in a lower number of patients included in the study. We reviewed these patients' records at least three times including paper records and electronic records to look for the missing data before excluding these patients and to minimize the risk of bias. A few patients did not undergo COVID-19 tests on the electronic system and were excluded from the final analysis. Microsoft Excel and the Statistical Package for the Social Sciences (SPSS) Statistics were used for data analysis. We analyzed variables associated with poor prognosis and prolonged hospital stay. Data was collected about patient demographics, comorbidities, and treatment received during hospital stay, and we performed the chi-squared test and Kaplan-Meier curve to measure survival analysis in patients with suspected COVID-19 admitted to hospital. We divided patients into COVID-19-positive and COVID-19-negative groups to compare the outcome and management of patients during admission and to measure survival analysis for both groups. 

## Results

Among 269 patients, only 153 were included in the study. The mean age of the COVID-19 and non-COVID-19 patients was 62.8 years and 65.9 years respectively. Male and female patients accounted for 47% and 53%, respectively, in the COVID-19 group, whereas male and female patients accounted for 51.6% and 48.4%, respectively, in the non-COVID-19 group. In terms of demographic distribution, the majority of the patients were white (57.4%), followed by Asians (19.1%) and Afro-Caribbean (10.6%), respectively, as shown in Table 1. There were no COVID-19 swabs for the seven patients included in the audit, but they were suspected to have COVID-19 based on clinical and radiological findings.

**Table 1:** Demographic distribution COVID-19: coronavirus disease 2019

		COVID-19	Non-COVID-19
Age (years)	Mean	62.8	65.9
	Median	65	69
Gender	Male	22 (46.8%)	48 (51.6%)
	Female	25 (53.2%)	45 (48.4%)
Ethnicity	White	27 (57.4%)	64 (68.8%)
	Asian	9 (19.1%)	10 (10.8%)
	Afro-Caribbean	5 (10.6%)	8 (8.6%)
	Others	6 (12.8%)	11 (11.8%)

**Figure 1:**
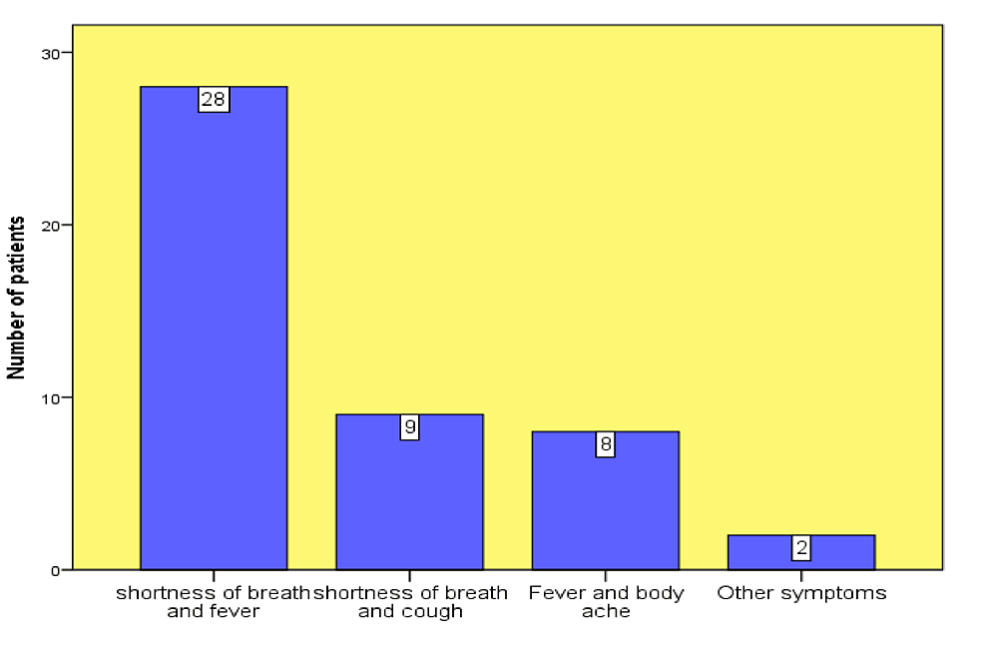
Clinical features of COVID-19 patients COVID-19: coronavirus disease 2019

**Figure 2:**
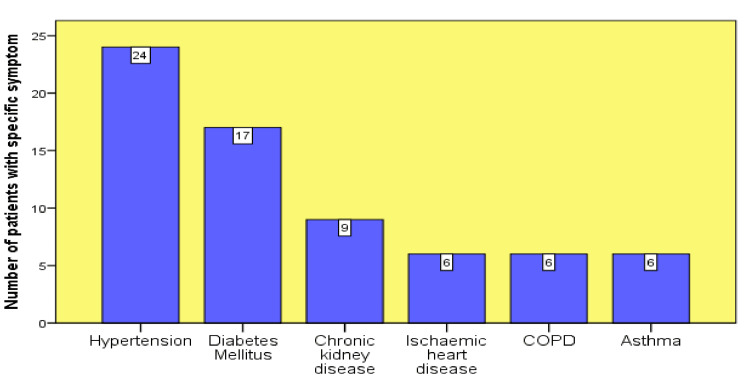
Comorbidities in COVID-19 group COVID-19: coronavirus disease 2019; COPD: chronic obstructive pulmonary disease

Most patients (n=28) presented with sob and fever, sob and cough (nine patients), and fever and body aches (eight patients). Only two patients experienced other symptoms, such as sore throat, chest tightness, rigors, and joint aches, as shown in Figure 1. Based on the comorbidities of patients admitted to the hospital during this time, HTN and DM were the major long-term illnesses, followed by chronic kidney disease. Most patients (n=24) had HTN, 17 had type 2 diabetes mellitus (T2DM), and nine had chronic kidney disease. Other comorbidities included asthma, ischemic heart disease (IHD), and chronic obstructive pulmonary disease (COPD), and six patients were affected by these comorbidities (Figure 2).

**Figure 3:**
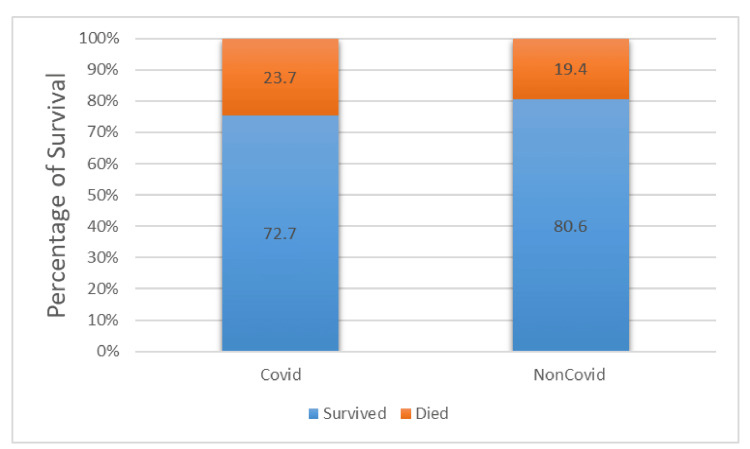
Percentage of survival

**Figure 4:**
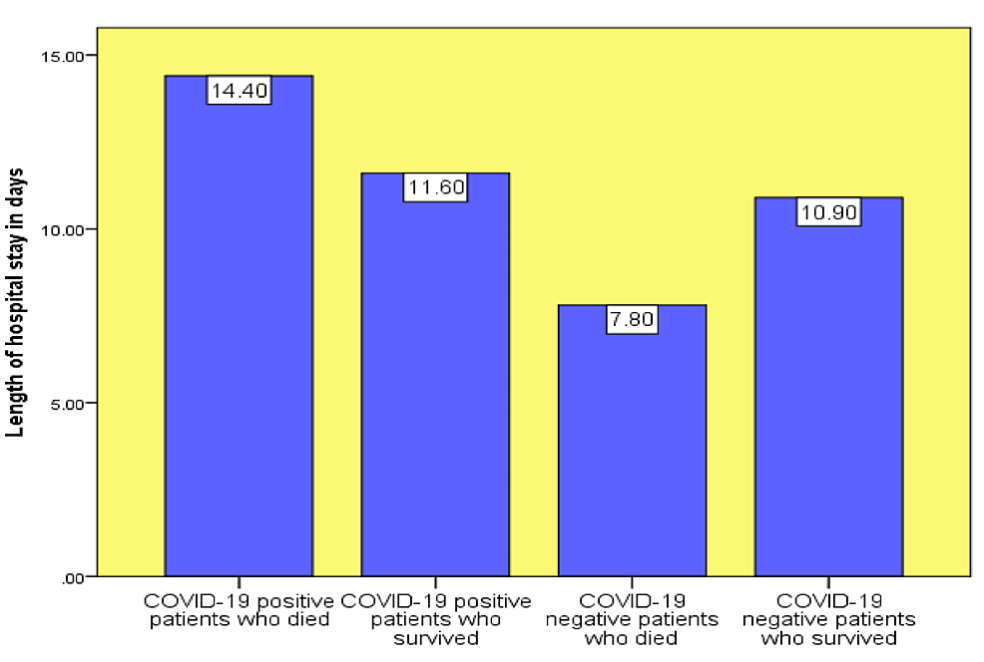
Length of stay in days for suspected COVID-19 patients The x-axis shows patient status and the y-axis shows the length of patient stay in days. COVID-19: coronavirus disease 2019

**Figure 5:**
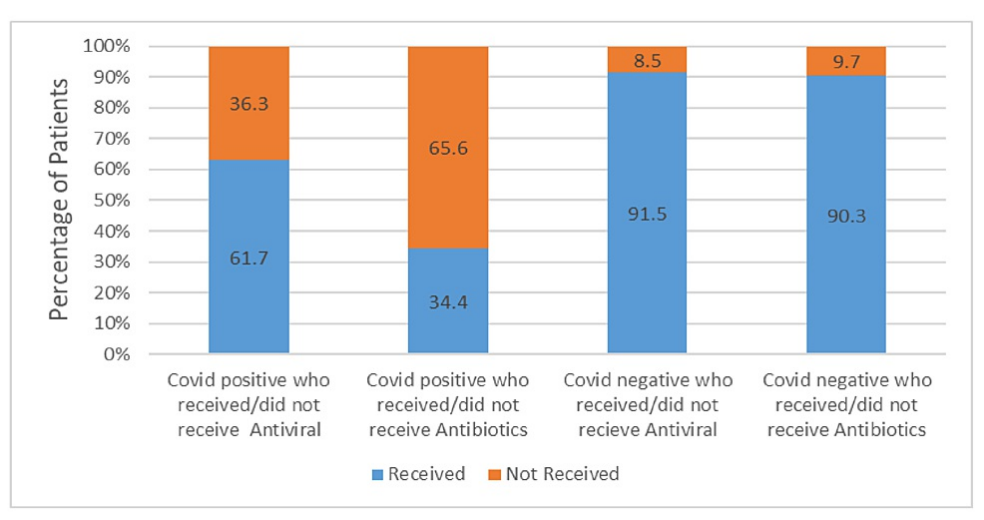
Percentage of patients who either received or did not receive antibiotics and antiviral therapy

Based on the survival analysis, more non-COVID-19 patients survived during hospital admission than did COVID-19 patients. Approximately 73% of the patients survived in the COVID-19 group compared to 81% in the non-COVID-19 group (Figure 3). Patients who were COVID-19 positive had longer hospital stays than those who were negative for the disease. The average length of stay was 14.4 days for COVID-19-positive survivors compared to 7.8 days for COVID-19-negative survivors (Figure 4). Based on the treatment received, approximately 62% of the COVID-19-positive patients received oseltamivir, whereas only one-third of this group (34.4%) received antibiotics during admission (Figure 5).

**Figure 6:**
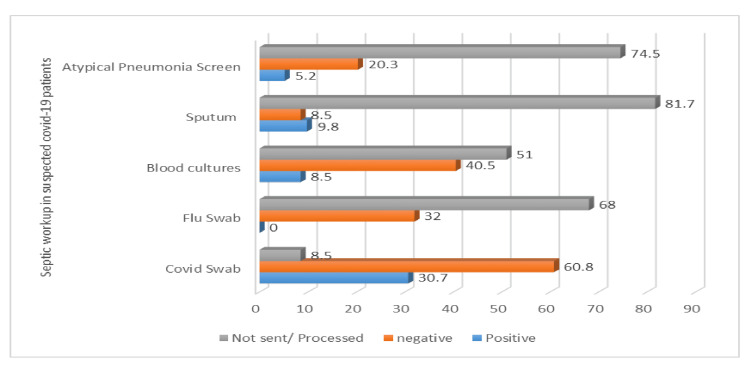
Sepsis workup in suspected COVID-19 patients COVID-19: coronavirus disease 2019

This could be because COVID-19 is considered primarily a viral illness, and antibiotics were not prescribed during the initial outbreak. On the other hand, over 90% of the patients in the non-COVID-19 group received both oseltamivir and antibiotics which could be because these patients were considered to have influenza and possible chest infections. Most patients (81.8%) admitted to the hospital had nasopharyngeal swabs sent for COVID-19 polymerase chain reaction (PCR), whereas 49% of patients had blood cultures and 32% of patients had flu swabs taken (Figure 6). Furthermore, 35.5% and 18.3% of the patients in both groups had atypical pneumonia screening and sputum cultures, respectively (Figure 6).

**Figure 7:**
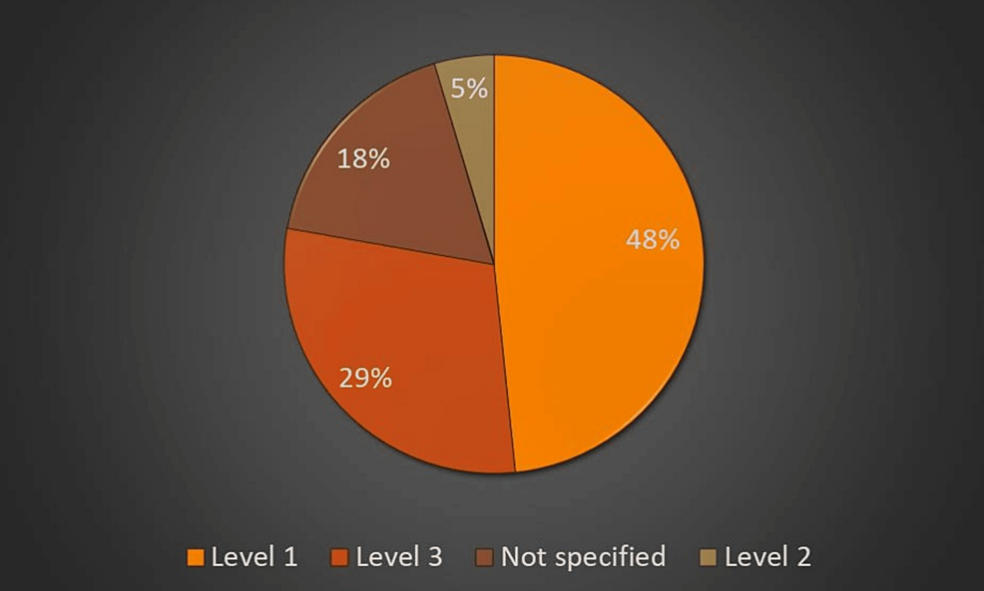
Treatment escalation plan for patients with suspected COVID-19 infection Level 1 care patients require acute medical ward care with input from critical care due to the risk of deterioration. Level 2 care patients require more regular monitoring including single-organ support. Level 3 care patients require respiratory and two or more organ support. COVID-19: coronavirus disease 2019

All patients admitted to the hospital during this period had an escalation plan in place. This was probably due to the fact that clinicians wanted to identify patients with poor prognoses and patients at risk of deterioration to make escalation plans in advance. We were not able to identify from the documentation discussions with families and how many of these patients' families were consulted about escalation plans. Figure 7 shows the treatment escalation plan for patients admitted to the hospital during this period. Numerous predictors of survival to hospital discharge have been identified. Chronic diseases play an important role in mortality and extended hospital stays. T2DM was the most common condition among COVID-19-positive patients, followed by HTN, acute kidney injury (AKI), elevated inflammatory marker levels, age, and organ failure. Patients with COVID-19 infection features in both lungs have poor outcomes (unilateral vs. bilateral, hazard ratio (HR) 8.32 (1.11-62.3), p=0.039) (Table 2). We performed the chi-squared test to check the association between various variables such as HTN, diabetes, acute kidney injury, accommodation, organ failure, inflammatory markers, and unilateral or bilateral COVID-19 features and outcomes.

**Table 2:** Predictors of the survival to hospital discharge in COVID-19 The chi-squared test was performed for these variables. COVID-19: coronavirus disease 2019; OR: odds ratio; CI: confidence interval; ACEi: angiotensin-converting enzyme inhibitor; ARB: angiotensin II receptor blocker; AKI: acute kidney injury; CRP: C-reactive protein

Characteristics	Characteristics outcome	OR	(99% CI)	P-value
Age (years)	>70 years	10.53	(1.017-1.091)	P=0.003
Accommodation	House/flat	1	(1.53-127)	P=0.019
	Nursing/residential home	13.9		
Hypertension	Absent	1	(1.27-10.6)	P=0.016
	Present	3.67		
Diabetes mellitus	Absent	1	(1.04-7.58)	P=0.042
	Present	2.81		
ACEi/ARB	Not taking	1	(1.07-7.93)	P=0.037
	Taking	2.91		
Location of COVID-19	Unilateral	1	(1.32-81.3)	P=0.031
	Bilateral	10		
Leukocyte count	Raised	12.5	(1.017-1.245)	P=0.022
AKI	Absent	1	(1.67-13.9)	P=0.004
	Present	4.82		
CRP	>100	1.008	(1.004-1.012)	P=0.0002
D-dimer	Elevated	1.416	(0.338-5.935)	P=0.634
Any organ failure	Single	1	(1.44-20.3)	P=0.012
	Multiple	5.42		

**Figure 8:**
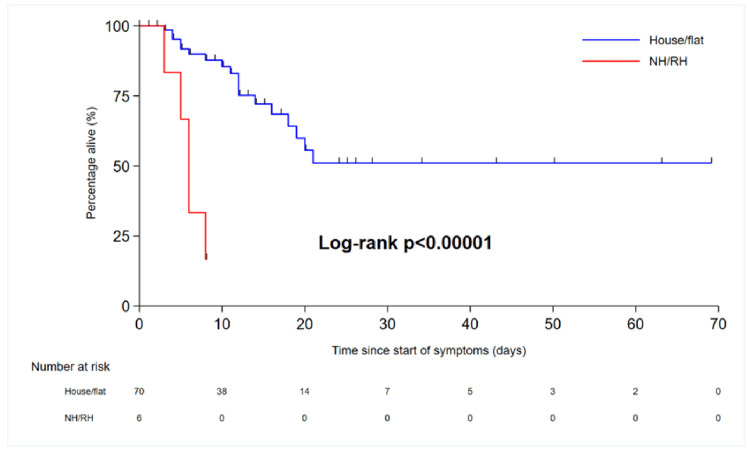
Kaplan-Meier curve showing overall survival in patients living in nursing homes/residential homes vs. house/flat

**Figure 9:**
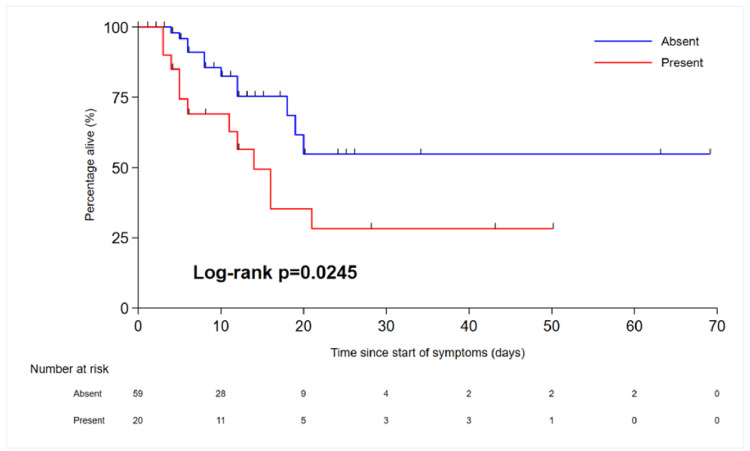
Kaplan-Meier curve showing overall survival in patients with AKI vs. without AKI AKI: acute kidney injury

**Figure 10:**
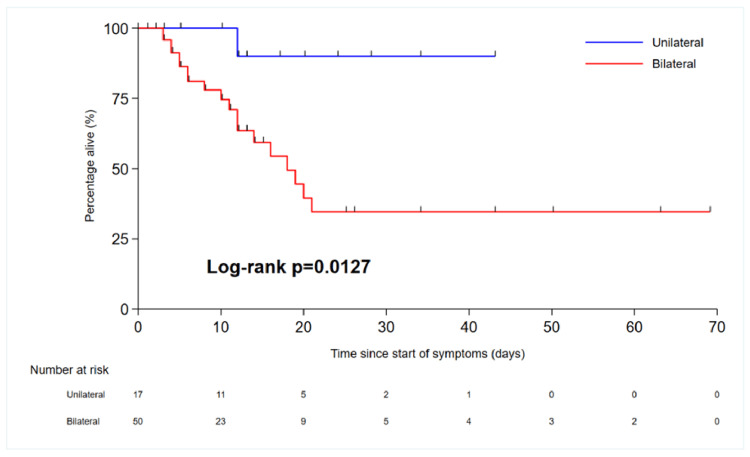
Kaplan-Meier curve showing overall survival in patients with unilateral COVID-19 vs. bilateral COVID-19 COVID-19: coronavirus disease 2019

Patients from nursing and residential homes had poorer outcomes than those from homes or flat homes. The HR for nursing home vs. house/flat was 9.68 (3.03-30.9), p=0.0001 (Figure 8). This is most likely because patients from nursing homes have more comorbidities and poorer reserves compared to patients living in their own homes. Additionally, patients living independently at home are likely to be more active than nursing home residents. Similarly, patients with AKI and COVID-19 had poorer outcomes and longer hospital stays than patients positive for COVID-19 but without AKI (Figure 9). Renal impairment was associated with poor outcomes in our study group, and similar findings have been reported in other national and international studies. Patients with COVID-19 infiltrates in both lungs on CT imaging had poor outcomes or higher HRs than patients with unilateral COVID-19 lung features (Figure 10). The overall survival of patients with COVID-19 is presented in the Kaplan-Meier curves (Figures 8-10).

## Discussion

The COVID-19 pandemic, which started in China and spread rapidly worldwide, has resulted in significant morbidity and mortality. The viral pathogen is highly virulent and can mutate, resulting in outbreaks worldwide. Patient stratification can be useful to identify patients at a higher risk of deterioration, and several studies have been conducted in this regard [1-7]. Several studies have assessed mortality predictors in various countries [16-18]. The main risk factors for mortality in COVID-19 patients reported by various studies include male sex, advanced age, obesity, HTN, coronary artery disease, DM, and malignancy [1,2]. Additionally, certain biochemical and physical signs are also related to higher mortality, including fever, hemoptysis, loss of consciousness, renal impairment, deranged liver function, and elevated levels of inflammatory biomarkers such as CRP, LDH, and procalcitonin. Our study also showed that patients with renal impairment, fever, sob, and raised inflammatory markers had poor outcomes compared to those without these, and similar data was published by the WHO that showed high mortality in Italy compared to China, which was attributed to male gender, advanced age, and comorbidities [2,19]. Similarly, there was a significant difference in mortality among Western countries, and Germany reported a very low fatality rate compared to the United Kingdom, Spain, France, and Italy [2,4]. The high mortality in these studies was attributed to old age, male gender, and comorbidities, and our study showed similar findings.

Bellan et al. reported that mortality was high among older patients, and arterial HTN was the most common comorbidity among these patients [2]. They also noted that coronary artery disease, HTN, malignancy, atrial fibrillation, chronic kidney disease, smoking, COPD, and dementia were associated with poor prognosis [2,3]. This study also reported an association between increased CRP and LDH levels, low platelet and lymphocyte counts, and mortality risk, and our study also showed similar results [2-5]. A previous study reported similar findings, and patients with severe pneumonia, multiorgan dysfunction, interstitial lung disease, heart failure, immunosuppressive conditions, COPD, male sex, malignancy, and sepsis were more likely to have poor outcomes [16].

España et al. performed a retrospective study on COVID-19 patients requiring hospital and ICU admission [18]. The study included patients from nursing homes (3567 patients) and the general population (15,201 patients) [18]. COVID-19 infection requiring hospital admission was associated with an increased mortality risk in both groups. Data from nursing home patients showed that male sex, old age >80 years, COVID-19 infections requiring hospital admission, cardiovascular disease, renal disease, and dementia were associated with increased mortality risk, and our study also showed similar findings. Anticoagulants and lipid-lowering therapy had protective effects in this group of patients in this study although we could not collect data regarding these two variables and were not able to compare this data. On the other hand, for patients in the general population group, predictors of mortality were male gender, age >60 years, cardiovascular disease, dementia, respiratory disease, liver disease, diabetes with end-organ damage, malignancy, COVID-19 infection requiring hospital admission, or hospital admission for any cause within the last month. Our study also showed that diabetes, renal disease, HTN, cardiovascular disease, increased D-dimer levels, being a nursing home resident, and old age were associated with higher mortality risk. Additionally, our study also showed that patients with COVID-19 features in both lungs were associated with a higher mortality risk than single lung features.

Several meta-analyses have previously reported an association between male sex, advanced age, comorbidities, and increased COVID-19 mortality risk; however, a major weakness of these studies is the heterogeneity in the data. Additionally, all of these studies have focused on hospitalized patients using laboratory data that lack uniformity [19-21]. Yang et al. reported a higher rate of hospital and ICU admission and mortality in patients with obesity and COVID-19 infections [21]. Comorbidities such as COPD, diabetes, chronic liver disease, cardiovascular disease, and malignancy were shown to be associated with a higher mortality risk among most patients. From these, diabetes is the most prevalent comorbidity seen among patients with COVID-19 infection, and although the exact etiology is unclear, an exacerbated pro-inflammatory cascade and impaired immune response are believed to be involved in this association [22-24]. Similarly, patients with underlying malignancy and chronic kidney disease are more susceptible to COVID-19, and both are associated with an increased mortality risk [25,26]. These studies also showed that certain medications, such as anticoagulants and statins, have protective roles against COVID-19 infection mortality, which could be due to the anti-inflammatory role of statins as COVID-19 is a pro-inflammatory condition. Anticoagulants are protective due to the prothrombotic nature of COVID-19; however, the use of both these medications is reduced in patients aged >85 years due to functional and cognitive decline [27-30]. We did not collect data regarding the use of anticoagulation and statin use in patients admitted to hospitals with suspected COVID-19 infection.

A study based on data from COVIDENCE UK, a prospective population-based UK study of acute respiratory infections (ARIs) in adults, demonstrated that both COVID-19 and non-COVID-19 infections related to severe acute respiratory syndrome coronavirus 2 (SARS-CoV-2 infection) were associated with increased symptoms and decreased health-related quality of life (HRQoL) [31]. Patients with COVID-19 infection-related ARIs were more likely to have problems with taste/smell, lightheadedness, or dizziness than those with non-COVID-19 ARIs [31]. Similarly, patients with COVID-19 were more likely to experience hair loss, unusual sweating, unexpected palpitations, and memory problems than non-COVID-19 ARI. Another study showed that patients with prolonged COVID-19 were more likely to have anterior pituitary deficiencies [32]. The prevalence of symptoms in patients with a long COVID-19 infection has been reported to range from low to 93% in this study [32]. The presence of endocrine abnormalities in acute COVID-19 infections has been reported previously; however, few studies have reported long-term endocrine problems in patients with long-term COVID-19 infections [32-34]. 

Limitations

There are several limitations to our study, including its retrospective nature, small sample size, and missing data for a significant number of patients, resulting in the exclusion of these patients from the study. We however made maximum efforts to retrieve this missing data before excluding these patients.

## Conclusions

COVID-19 is associated with higher mortality and morbidity, and our study showed that certain conditions and variables are associated with a higher mortality risk. Our study showed that male sex was associated with a higher mortality risk and patients with comorbidities were more likely to have a poor prognosis. Patients with diabetes and cardiovascular disease are more likely to have poor outcomes. Nursing or care home residents were at an increased risk of mortality compared to patients from homes and flats. Advanced age was associated with poor outcomes in the study group. These data can be useful for planning future health interventions for cases of disease outbreaks.
